# Cell walls of filamentous fungi – challenges and opportunities for biotechnology

**DOI:** 10.1007/s00253-025-13512-3

**Published:** 2025-05-24

**Authors:** Katharina J. Ost, Mounashree Student, Stefan Cord-Landwehr, Bruno M. Moerschbacher, Arthur F. J. Ram, Mareike E. Dirks-Hofmeister

**Affiliations:** 1 Laboratory for Food Biotechnology, Faculty of Agricultural Sciences and Landscape Architecture, Osnabrück University of Applied Sciences, Oldenburger Landstraße 62, 49090 Osnabrück, Germany; 2 Institute for Biology and Biotechnology of Plants, University of Münster, Schlossplatz 8, 48143 Münster, Germany; 3 Fungal Genetics and Biotechnology, Institute of Biology Leiden, Leiden University, Sylviusweg 72, 2333 BE Leiden, The Netherlands

**Keywords:** Fungal cell walls, Biotechnology, Filamentous fungi, Industrial enzymes, Chitin and chitosan

## Abstract

**Abstract:**

The cell wall of filamentous fungi is essential for growth and development, both of which are crucial for fermentations that play a vital role in the bioeconomy. It typically has an inner rigid core composed of chitin and beta-1,3-/beta-1,6-glucans and a rather gel-like outer layer containing other polysaccharides and glycoproteins varying between and within species. Only a fraction of filamentous fungal species is used for the biotechnological production of enzymes, organic acids, and bioactive compounds such as antibiotics in large amounts on a yearly basis by precision fermentation. Most of these products are secreted into the production medium and must therefore pass through fungal cell walls at high transfer rates. Thus, cell wall mutants have gained interest for industrial enzyme production, although the causal relationship between cell walls and productivity requires further elucidation. Additionally, the extraction of valuable biopolymers like chitin and chitosan from spent fungal biomass, which is predominantly composed of cell walls, represents an underexplored opportunity for circular bioeconomy. Questions persist regarding the effective extraction of these biopolymers from the cell wall and their repurposing in valorization processes. This review aims to address these issues and promote further research on understanding the cell walls in filamentous fungi to optimize their biotechnological use.

**Key points:**

*• The highly complex cell walls of filamentous fungi are important for biotechnology.*

*• Cell wall mutants show promising potential to improve industrial enzyme secretion.*

*• Recent studies revealed enhanced avenues for chitin/chitosan from fungal biomass.*

**Graphical Abstract:**

**Figure:**
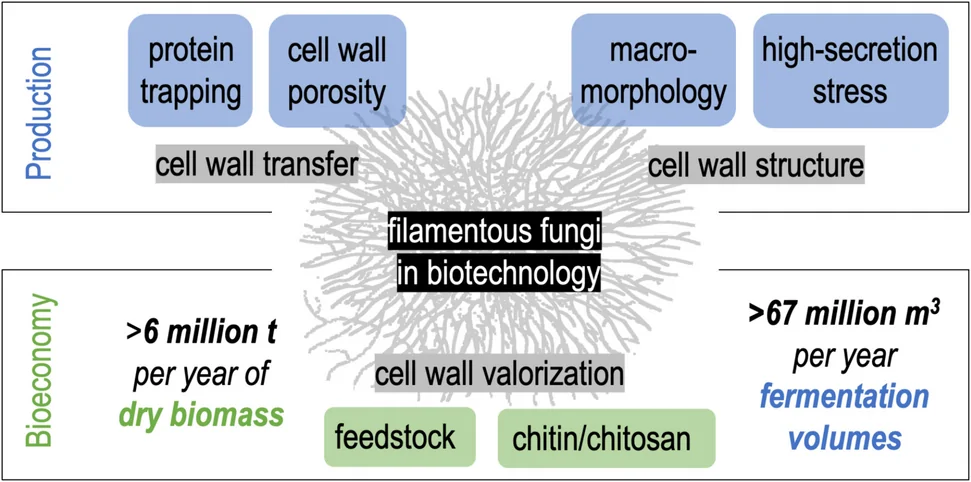

## Introduction

Many bacteria and yeasts can be easily grown in fermenters to produce commercially interesting products, and large-scale fermentation volumes are used to profit from economies of scale (Demain and Adrio 2008; De Brabander et al. 2023). But sometimes, the metabolism, genetic repertoire, or posttranslational modification potential of prokaryotes and unicellular eukaryotes such as yeasts are not complex enough for target compound production. Therefore, filamentous fungi are used to produce various commodities and specialty products (Nielsen et al. 1995; Meyer 2008; Meyer et al. 2020). Moreover, filamentous fungi outcompete bacteria and yeasts in terms of efficiency of protein secretion, reaching levels of secreted proteins of up to 100 g/L, with the target protein secreted and present in the spent medium (Ward 2012; Cairns et al. 2018). The main filamentous fungal organisms used for biotechnological production are *Aspergillus niger*, *Aspergillus oryzae*, *Trichoderma reesei*, *Penicillium chrysogenum*, *Rhizopus* sp., and *Rhizomucor miehei* (Table 1). Every year, millions of tons of fungal cells are produced in commercial biotechnological processes via (precision) fermentation (Allied Market Research 2024; Valuates Reports 2024). Valuable chemicals, food enzymes, antibiotics, and organic acids are produced and secreted, thus requiring passage through the fungal cell wall. In attempts for strain improvement the cell wall and cell wall-related genes increasingly appear as targets. At the same time, cell walls occur as an important by-product in high amounts after fermentation as part of the separated fungal biomass (Nikkilä et al. 2020), and cell wall components are considered as potentially interesting chemical building blocks. Thus, in the interest towards a circular bioeconomy, these biotechnological processes give the chance of recovering and valorizing cell wall components of high purity and reproducibility (Meyer et al. 2016, 2020; Grimm and Wösten 2018; Cairns et al. 2018, 2021b; Wösten 2019).

**Table 1:** Estimated yearly production and fermentation volumes of major industrial products derived from filamentous fungi fermentation. The source of the underlying data is explained in the footnotes below the last table row

Fungal species as production organism		Product	Annual production (commercial product)	Share of fungal species from the whole production	Assumed content of the ingredient in the products	Annual production of the ingredient by the fungus	Assumed yields of ingredients in industrial fermentations	Annual fermentation volumes
			[× 1000 t/a]			[× 1000 t/a]	[t/m^3^]	[× 1000 m^3^/a]
*Ascomycetes*	Aspergillus sp.	Citric acid	3,300 ^a^	80% ^b^	100%*	2,640	0.11^c^	24,000
		alpha-Amylases	25,500 ^d^	10% *	10% ^e^	255	0.03 ^f^	8,500
		Glucoamylase	8,500 ^g^	85% ^h^	10% ^e^	765	0.03 ^f^	25,500
		Cellulases	549 ^i^	20% *	10% ^e^	11	0.03 ^f^	366
		Chymosin	n.r	50% ^j^	n.r	n.r	n.r	1,500 ^k^
	*Penicillium* sp.	Penicillin G	60 ^l^	95% *	100% ^e^	57	0.07 ^m^	814
	*Trichoderma* sp.	Cellulases	549 ^i^	70% *	10% ^e^	38	0.10 ^n^	385
*Mucoromycetes*	*Rhizopus *sp.	Glucoamylase	8,500	15% ^h^	10% ^e^	85	0.03 ^f^	2,833
	*Rhizomucor *sp.	Microbial rennet	n.r	99% ^k^	n.r	n.r	n.r	2,970^k^
								66,686

*n.r.* not relevant to our calculations

^*^Estimated values

^a^Książek (2024)

^b^Vandenberghe et al. (2017)

^c^Kristiansen et al. (1998) stated that citric acid constitutes up to 15% of the total production, whereas Max et al. (2010) postulated final yields of 110–140 g/L of citric acid; to keep it conservatively, we calculated with 11 tons per m^3^

^d^The proportion of microbially produced alpha-amylases is estimated to be 25–30% of the industrial enzyme market (Reddy et al. 2003; de Souza and de Oliveira e Magalhães 2010; Balakrishnan et al. 2021). Based on the growing market (Allied Market Research 2024), we assume 30% for our calculations

^e^Most industrial enzyme products are standardized to a minimal enzyme activity but mostly state a minimal protein content of 10%

^f^Cairns et al. (2018) stated that industrial *Aspergillus* strains produce 30 g/L of extracellular enzyme; thus, we used a quotient of 0.03 to calculate the fermentation volume from the annual production

^g^The number was concluded from the Astute Analytica (2024) market research report on “Enzyme Market,” which stated that in 2023, the world total enzyme production by microorganisms was 85 million tons. Mendonça et al. (2023) estimated the share of glucoamylase in the industrial enzyme market as 10%

^h^Norouzian et al. (2006) mentioned *Aspergillus* and *Rhizopus* to be the only industrially relevant fungal glucoamylase producers, so we assumed a ratio of 85:15 for these two species

^i^The forecast was taken from the Global Cellulase Market Research Report from Valuates Reports (2024)

^j^Sutay Kocabaş et al. (2022) stated that fermentation-produced chymosin is either produced by *Aspergillus* (Chr. Hansen) or *Kluyveromyces* (DSM)

^k^Guerrand (2018) postulated that Chr. Hansen holds 35% of the market share for dairy products and DSM 25–30% including enzymes ($250 million per year) and 60% of the market of dairy enzymes to be milk clotting enzymes (= $150 million per year). Additionally, the authors estimated the cost of rennet enzymes to be approximately $0.05 per liter. From these values, we concluded that the total fermentation volume for rennets was 3,000 million liters, or 3 million m^3^ per year

^l^Thykaer and Nielsen (2003) stated that the annual production of penicillin exceeds 60,000 tons

^m^Demain (2006) stated commercial *Penicillium* strains to produce 70 g/L penicillin

^n^Ward (2012) reported that *Trichoderma reesei* secretes 100 g/L of cellulase

To provide a tangible impression of the annual quantities of fermentation volumes and fungal cell wall biomass from processes using filamentous fungi from different areas of biotechnology, we accumulated data to come to an approximate estimate as given in Table 1. Considering only fermentation products with the highest estimated yearly production, such as citric acid (Kristiansen et al. 1998; Max et al. 2010; Vandenberghe et al. 2017; Książek 2024), alpha-amylase (Reddy et al. 2003; de Souza and de Oliveira e Magalhães 2010; Balakrishnan et al. 2021; Allied Market Research 2024), glucoamylase (Norouzian et al. 2006; Mendonça et al. 2023) and cellulase enzymes (Ward 2012; Paloheimo et al. 2016; Valuates Reports 2024), penicillin G (Thykaer and Nielsen 2003; Demain 2006; Wenzel and Müller 2013), chymosin, and microbial rennets (Guerrand 2018; Sutay Kocabaş et al. 2022), the yearly fermentation volumes of filamentous fungi can be estimated to exceed 67 million cubic meters. The resulting dry biomass can be assumed to be 4–14% of the fermentation volume, depending on the strain and process (Koutinas et al. 2003; Xie et al. 2020; Bodie et al. 2021), and can then be estimated to sum up to 3–9 million tons of dry weight per year. To date, these side streams are usually either wasted or used in agriculture as fertilizers or feed (Das et al. 2002; Grimm and Wösten 2018; Nikkilä et al. 2020; Meyer et al. 2020; Cairns et al. 2021b; Crosino et al. 2021).

From a biological perspective, hyphal mycelia produced by filamentous fungi are not ideally suited for submerged fermentation. Filamentous hyphae are sensitive to shear stress caused by stirring (Amanullah et al. 2002) and can affect free nutrient diffusion (Olsvik and Kristiansen 1994; El Enshasy 2022). Furthermore, filamentous fungi growing in submerged cultures tend to develop different morphological phenotypes, ranging from dispersed growth to compact pellets, which significantly challenges industrial performance and handling (Grimm et al. 2005; Veiter et al. 2018; El Enshasy 2022). Filamentous fungi are evolutionarily optimized to grow on solid media, developing hyphal tip growth propelled by high turgor pressure (Papagianni 2004; Lew 2019). The cytosol of filamentous fungi tends to be concentrated at the hyphal tips, whereas distant parts are typically empty or filled with vacuoles. The entire mycelium is surrounded by complex cell walls, which are material- and energy-intensive to build. In biotechnological applications, these cell walls influence product secretion and ultimately result in unused and costly biomass waste.

The objective of this review is to discuss the importance of fungal cell walls, particularly those of industrially relevant filamentous species, in biotechnology. This aspect with elaboration on cell walls in the context of industrial utilization of fungi, puts significant novelty in our attempt compared to previous review articles. We (i) briefly describe the current understanding of fungal cell wall composition, architecture, complexity, and flexibility, focusing on biotechnologically relevant filamentous fungal species from different taxonomic groups, excluding yeasts. We then (ii) summarize the fungal cell wall biosynthesis and assembly, remodeling, and integrity pathways, as well as up-to-date data on secretory pathways in fungi. Third, we (iii) focus on the assumably high impact of the cell wall on protein adsorption, morphology, high-secretion stress, and over-all enzyme productivity. Finally, we (iv) will discuss existing and future opportunities and challenges in utilizing fungal biomass, in particular the cell walls, most prominently as an alternative source of the valuable biopolymers chitin and chitosan.

## The fungal cell wall

Bacterial cell walls are rather rigid structures that essentially consist of a single, covalently linked peptidoglycan super-molecule enclosing the entire prokaryotic cell (Garde et al. 2021). In contrast, fungal and plant cell walls are more complex and flexible, where different types of polysaccharide polymers are connected by few covalent cross-links and many weak interactions, such as hydrogen bonds. In most plant cell walls, long cellulose fibrils are crosslinked by xyloglucans, and this alkali-insoluble fibrillar network is embedded in an alkali-soluble gel-like matrix composed of hydrated pectins (Cosgrove 2024).

A similar fiber-in-a-matrix architecture has also been suggested for fungal cell walls, as an alkaline insoluble core structure remains after extraction of an alkaline-soluble matrix (Wessels and Sietsma 1981). There is also a strong similarity in the physical structure of the insoluble fibers observed under an electron microscope, as well as in the chemical structure of chitin and cellulose molecules and crystals (Kostag and El Seoud 2021; da Rosa et al. 2024). However, recent developments in analytical techniques, such as enzymatic-mass spectrometric fingerprinting (EMS-FP) and, above all, solid-state nuclear magnetic resonance (ssNMR), which allow investigation of the native cell wall in situ without prior extraction of individual cell wall components, and the increasing possibility of generating genetically engineered mutants, have provided evidence that tends to challenge this assumption of a simple fiber-in-matrix-architecture (Gow et al. 2017; Chakraborty et al. 2021; Fernando et al. 2023; Sun 2024). However, detailed analyses have only been performed on a few filamentous fungi, including the ascomycetes *Aspergillus fumigatus* and some other Aspergilli, the basidiomycetes *Schizophyllum commune* and *Cryptococcus neoformans*, and a few chitosan-containing mucoromycetes, such as *Rhizopus delemar* (Kang et al. 2018; Ehren et al. 2020; Safeer et al. 2023; Cheng et al. 2024). These results show that fungal cell walls exhibit tremendous variability both between species and within a given species under different environmental conditions or at different developmental stages. Clearly, any generalizations based on the too few examples available today are necessarily preliminary and need to be taken with great caution.

**Fig. 1:**
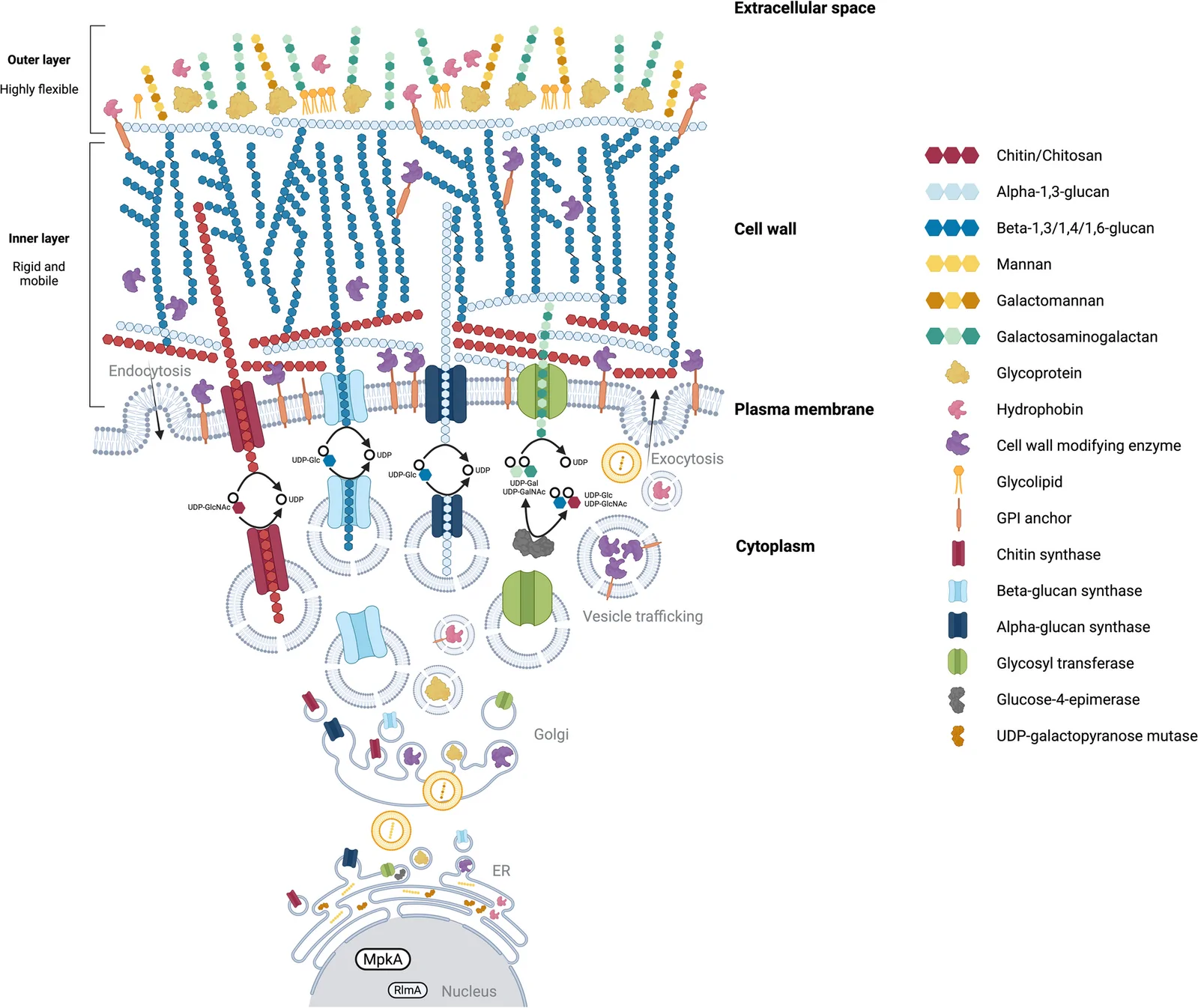
Schematic representation of fungal cell wall structures and biosynthetic proteins. UDP: uridine diphosphate; Glc: glucose; Gal: galactose; GlcNAc: *N*-acetylglucosamine; GalNAc: *N*-acetylgalactosamine; ER: endoplasmic reticulum; RlmA: MADS-type transcription factor; MpkA: mitogen-activated protein kinase A; GPI: glycosylphosphatidylinositol (created using BioRender.com and modified from Yoshimi et al. 2016; Kang et al. 2018; Speth et al. 2019; Fontaine and Latgé 2020; Gow and Lenardon 2023)

Figure 1 and the remaining of this section provide our view on the current understanding of fungal cell wall composition, architecture, synthesis, and assembly. As mentioned above, we are fully aware that this working model of the fungal cell wall is a snapshot of this work in progress.

### Restrictions and improvement of cell wall analytics

As mentioned before, conventional cell wall analysis of fungi and other organisms historically relied on chemical or enzymatic hydrolysis of the polymers and analysis of the degradation products, often preceded by sequential extraction of different polymers (Heux et al. 2000; Wu et al. 2005; Cai et al. 2006; Krake et al. 2024). These techniques have yielded an inventory of cell wall polymers with known monomer compositions and their glycosidic linkages, but have struggled to resolve their dynamic multilayered architecture (Latgé 2007; Blatzer et al. 2020). Thus, the interactions of polymers to form the complex architecture of the cell wall remain largely speculative. Furthermore, these analyses are prone to errors due to material loss during harsh chemical treatment and/or modifications introduced during the process.

Recent advances in high-resolution techniques, such as ssNMR, have more accurately mapped biomolecular interactions in the cell walls of *A. fumigatus, S. commune, Rhizopus*, and *Mucor* species, revealing significant deviations from earlier cell wall models (Kang et al. 2018; Ehren et al. 2020; Chakraborty et al. 2021; Ghassemi et al. 2022; Safeer et al. 2023; Lamon et al. 2023; Cheng et al. 2024). However, also reports on polysaccharide and protein composition, as well as the crosslinking of cell wall polysaccharides, differ significantly in the literature, and it remains to be seen whether these differences are the result of the method of analysis, different growth conditions, or proof of the immense flexibility of fungal cell walls.

EMS-FP has helped fine-tune cell wall analyses, for example, providing initial insights into the distribution of acetylated and deacetylated monomeric units within chitin (a polymer of *N*-acetylglucosamine) and chitosan (partially deacetylated chitin) chains (Urs et al. 2023). Solid-state NMR on intact cell walls is well suited for revealing polymer configurations and polymer–polymer interactions, and this has resulted in the realization of additional complexities, such as the existence of up to a dozen subtypes of chitin crystal structures (Cheng et al. 2024). Clearly, advanced cytochemical probes such as antibodies, lectins, or biotechnologically produced affinity proteins, as well as advanced microscopic techniques such as solid-state dynamic nuclear polarization (DNP-) NMR, which has a sensitivity 100–1000 times higher compared to conventional ssNMR, mass spectrometry imaging (MSI), or super-resolution and live-cell imaging techniques such as direct stochastic optical reconstruction microscopy (dSTORM), structured illumination microscopy (SIM), or confocal laser scanning microscopy (CLSM), will contribute to further refining the spatial resolution of fungal cell wall analysis in the near future (Lin et al. 2016; Islam et al. 2017; Götz et al. 2020).

### Cell wall components, biosynthesis, and dynamic remodeling

Typically (Fig. 1), fungal cell walls are believed to have two layers: an evolutionary apparently rather conserved rigid skeletal layer of chitin, beta-1,3-glucans, beta-1,6-glucans, and beta-1,3-beta-1,6-glucans and a more variable, partially gel-like and sometimes hydrophobic outer layer varying between and within species that may include alpha-/beta-glucans and other homo- and heteropolysaccharides as well as (phosphor)glycoproteins, hydrophobins, or melanin, etc. (Latgé 2007, 2010; de Groot et al. 2009; Beauvais et al. 2014; Beauvais and Latgé 2015; Erwig and Gow 2016; Gow et al. 2017; Yoshimi et al. 2017; Ehren et al. 2020; Safeer et al. 2023). This outer layer is probably responsible for mediating interactions with the environment, including a stealth mechanism of pathogenic fungi to cloak the conserved rigid core, thus preventing its recognition by the immune systems of their hosts (Gow et al. 2017; Garcia-Rubio et al. 2020; Student et al. 2024). Studies on the cell wall structure, biosynthesis, and remodeling mechanisms are important, given that cell walls have long been recognized as an ideal target for antifungal therapies, as fungal cell wall polymers are unique and do not occur in humans or, with few exceptions, in plants.

Fungal cell wall polymers are synthesized and assembled through tightly regulated enzymatic pathways in the cytoplasm or at the plasma membrane at the hyphal apex where they integrate into the growing wall (Yoshimi et al. 2016; Kang et al. 2018; Garcia-Rubio et al. 2020). Covalent crosslinks between polymers must be formed extracellularly, possibly by GPI-anchored enzymes on the outer surface of the plasma membrane (Hopke et al. 2018). While some polymers, such as chitin, alpha-1,3-glucan, and beta-1,3-glucans are known to be synthesized by transmembrane glycosyl transferases and directly transported into the extracellular space (Brauer et al. 2023), other cell wall polymers are thought to be synthesized in vesicles and secreted into the cell wall by exocytosis (Henry et al. 2016), but this is more an assumption than supported by experimental evidence. Studies in *Ustilago maydis* and *Zymoseptoria tritici* have shown that chitin synthase and beta-glucan synthase co-localize in the same vesicle which are transported along microtubules to the growing hyphal tip (Schuster et al. 2016, 2020). These studies highlight that the microtubule-dependent co-delivery of cell wall enzymes is a conserved mechanism in both basidiomycetes and ascomycetes, contributing to the efficient synthesis of the fungal cell wall.

Chitin is located in the inner layer and enhances the structural stability and resistance to enzymatic degradation (Bowman and Free 2006; Free 2013; Brown et al. 2019; Garcia-Rubio et al. 2020). Various chitin synthases (Chs) produce chitin polymers with different lengths at specific sites (Mellado et al. 1996; Brauer et al. 2023). Cell wall beta-glucan forms a crucial network for cell shape and mechanical strength and is produced by plasma membrane-bound beta-glucan synthases (Fks) (Beauvais et al. 2001; Ruiz-Herrera and Ortiz-Castellanos 2019; Wagener et al. 2020; Hu et al. 2023). Some fungi also harbor *ags* genes encoding alpha-glucan synthases (Ags), which contribute to both the mobile outer cell wall structure and the inner structural stability (Yoshimi et al. 2017; Kang et al. 2018; Blatzer et al. 2020; Ehren et al. 2020; Safeer et al. 2023). Additionally, glycoproteins covalently bound to beta-glucans are vital for the cell wall structure and function, serving as adhesins or receptors for environmental and cellular interactions (Vogt et al. 2010; De Groot et al. 2013; Gow et al. 2017; Garcia-Rubio et al. 2020). Other complex polysaccharides with different structures represent cell wall mannans, such as glucomannans, galactomannans (GM), and galactoxylomannan (GXM), and galactosamins and galactosaminogalactans (GAG). These complex polysaccharides were found particularly in opportunistic fungal pathogens from *Aspergillus*, *Candida*, or *Cryptococcus* (Gow et al. 2017), and heterogalactans such as mannogalactans in the cell wall of *S. commune* (Dalonso et al. 2021). Genes involved in the synthesis of most of these polymers have been identified (Wang et al. 2013; Henry et al. 2016, 2019; Onoue et al. 2018; Speth et al. 2019; Hira et al. 2020; Fontaine and Latgé 2020; Kadooka et al. 2022). Cell wall (gluco)mannoproteins can cover the cell wall surface and are shown to be covalently bound to the cell wall core (Klis et al. 2007; Latgé 2007; Tefsen et al. 2012; Henry et al. 2019). In addition to (gluco)mannoproteins, also GAG, produced by the membrane-located glycosyl transferase Gbt3, which uses products from the cytosolic glucose-4-epimerase Uge3, is a recently discovered exopolysaccharide in the outer layer of the *A. fumigatus* cell wall that plays a key role in fungal interactions with the environment and the host immune system by forming a biofilm (Gravelat et al. 2013; Lee et al. 2014; Speth et al. 2019; Le Mauff 2020; Lamon et al. 2023; Le Mauff and Sheppard 2023). And these are just a few examples; it is not meant as a comprehensive overview of all the many complex polysaccharides reported in fungal cell walls.

The ability of fungi to thrive in various environments depends on the integrity and flexibility of their cell walls. To ensure structural stability while facilitating growth and adaptation, fungi employ sophisticated regulatory mechanisms, including the so-called cell wall integrity (CWI) pathway and the mechanisms and enzymes involved in cell wall remodeling (Beauvais et al. 2014; Beauvais and Latgé 2015). The CWI signaling pathways are vital for sensing and responding to cell wall stress. Mechano-sensor proteins, such as wall stress component-type sensors (WscA, WscB), detect changes in the cell wall and initiate signaling cascades to regulate the expression of genes related to cell wall biosynthesis (Rodicio et al. 2008; Yoshimi et al. 2022). These sensors are linked to intracellular signaling pathways, such as the mitogen-activated protein kinase (MAPK) cascade, including MapkA pathways, which are vital for stress signal transduction (Levin 2005, 2011; Perez and Cansado 2010; Dichtl et al. 2012, 2016; Park et al. 2016). Cell wall-modifying enzymes, including glucanases, chitinases, transglycosylases, mannosidases, peroxidases, deacetylases, and synthases (for chitin, glucans, or other cell wall components), play critical roles in the degradation and remodeling of fungal cell walls and are activated through transcriptional regulators such as RlmA (Damveld et al. 2005; Rocha et al. 2016).

The CWI and cell wall remodeling pathways work together to regulate cell wall biosynthesis, ensuring proper construction, maintenance, and adaptation of the fungal cell wall in response to environmental and physiological changes.

### Mechanism of protein secretion and transfer across the fungal cell wall

The ability of filamentous fungi to secrete large amounts of enzymes into the environment is the key reason for their successful application in industrial biotechnology. Their superior secretion performance originates from their lifestyle, with secreted enzymes and organic acids enabling nutrient digestion and absorption from their surroundings (Nevalainen and Peterson 2014; McCotter et al. 2016). Protein secretion in filamentous fungi – as in all organisms – is a complex and highly regulated process involving protein biosynthesis, post-translational modifications, and transport to the extracellular environment (reviewed by Sakekar et al. 2021 and Jadhav et al. 2024). Moreover, the filamentous growth of fungi adds extra complexity because of the relatively long distances for secretion vesicles to be transported to the growing tip of the hyphal cell (Takeshita 2016; Cairns et al. 2021b).

The protein secretion process in filamentous fungi follows the conventional secretion pathway using the ER-Golgi route: extracellular proteins are synthesized at ribosomes, translocated into the ER lumen via an N-terminal signal sequence, folded, and modified post-translationally within the ER (Boppidi et al. 2018; Wang et al. 2020; Jadhav et al. 2024). Folded proteins are packaged into vesicles and sent to the Golgi apparatus for further processing, including sorting (Nevalainen and Peterson 2014; Jadhav et al. 2024). Vesicles with mature secretory proteins or cell wall-remodeling enzymes travel from the Golgi along microtubules and actin filaments to the Spitzenkörper, releasing their contents into the extracellular space upon fusion with the plasma membrane. The classical secretion efficiency is usually much higher for native fungal proteins than for heterologous proteins, often by a factor of 10–1000 (Boppidi et al. 2018; Sakekar et al. 2021). *A. niger* can generate more than 25–30 g/L of homologous glucoamylase in fermentation media (Cairns et al. 2018), while *T. reesei* can secrete up to 100 g/L of its cellulase (Ward 2012), demonstrating their exceptional productivity. More examples of highly valued heterologous protein production in fungal hosts have been reviewed by Punt et al. (2002), Wei et al. (2021), and Liu et al. (2023). For this reason, filamentous fungi have emerged as versatile platforms for industrial protein production. High levels of protein secretion have been linked to increased biomass formation and altered growth patterns (Hayakawa et al. 2011; Read 2011; Benoit et al. 2015; Fiedler et al. 2018b; Cairns et al. 2019a, 2021a).

Alternative unconventional secretion routes exist next to the conventional secretion pathway, providing a potential alternative for protein export. These unconventional protein secretion (UPS) routes bypass ER quality control and Golgi modifications (Stock et al. 2012). In filamentous fungi, an example of a UPS protein is the chitinase Cst1 in *U. maydis*, which follows a lock-type, unconventional, and signal peptide-independent secretion during cytokinesis (Stock et al. 2012; Reindl et al. 2019; Aschenbroich et al. 2019; Sakekar et al. 2021). Cts1 is crucial for cell wall biosynthesis and regulation, as it hydrolyzes chitin into smaller oligosaccharides for recycling or incorporation into the cell wall matrix (Langner and Göhre 2016). The use of unconventional secretion to co-export heterologous proteins into the culture supernatant is considered an attractive alternative for overcoming the limitations of conventional secretion pathways (Hayakawa et al. 2011; Read 2011; Terfrüchte et al. 2017; Kwon et al. 2017; Cairns et al. 2021a; Liu et al. 2023; Jadhav et al. 2024). In *A. niger*, the aspartic protease PepN has also been suggested to be secreted by an unconventional secretion pathway (Burggraaf et al. 2016). However, the molecular mechanisms underlying the UPS pathway in filamentous fungi are not yet fully understood.

After passing across the cell membrane through any secretory pathway, the secreted fungal proteins still need to pass through the cell wall. Studies on isolated fungal cell walls showed a permeability of 5 to 270 kDa (Trevithick and Metzenberg 1966; De Nobel and Barnett 1991; Jacobson and Ikeda 2005) depending on the species, its degree of melanization, and the fungal stress levels. It has been shown that fungi also have a specific extracellular vesicle transport for macromolecules, including proteins, via the cell wall, which is highly similar to mammalian exosomes and bacterial vesicles (Casadevall et al. 2009; Oliveira et al. 2010; Rodrigues et al. 2015). Although the exact mechanism by which vesicles pass through the cell wall remains unclear, researchers have proposed various potential and partly controversial explanations, such as the existence of pores or channels (De Nobel and Barnett 1991; Casadevall et al. 2009; Rodrigues et al. 2015). Chevalier et al. (2023) describe *A. nidulans* cell walls as dynamic and stable, balanced between secretion and expansion. They showed that localized remodeling occurs at the hyphal tip through secretion of enzymes that assemble and soften the cell wall (Chavelier et al. 2023). This remodeling is spatially confined and regulated via mechanical feedback, where wall deformation promotes vesicle recruitment, enabling hyphal expansion at the apex. The authors reported that the cell wall at the hyphal apex is thinner and softer than the lateral wall, suggesting that this apical region forms a mechanical barrier through which vesicles can pass. Local softening and decreased thickness correlate with secretory vesicle accumulation, likely facilitating vesicle docking and protein interactions with the cell wall matrix. Further, de Paula et al. (2019) reported that extracellular vesicles transport cellulases in the industrially relevant enzyme producer *Trichoderma reesei*, which shows that vesicle transport via the cell wall is not limited to a communication system for pathogenic species, such as *Cryptococcus* sp., *A. fumigatus*, and *Candida albicans*, but could be a common system in fungi. The extent to which unconventional protein secretion and vesicle transport via the cell wall play a crucial role in high-level industrial protein secretion is not yet evident in the literature; however, it is worth further investigation.

## How fungal cell wall structure affects industrial enzyme production

Most industrial hyperproduction strains have historically originated from classical strain engineering without any targeted genetically engineered modifications (Nevalainen and Peterson 2014). For example, high levels of cellulase expression have been generated by random mutagenesis and selection, resulting in *T. reesei* strain Rut-C30 being the parental strain of industrially used *Trichoderma* cellulase producers (Eveleigh and Montenecourt 1979). The industrial Aspergilli separate genetically in acid and enzyme producing strain lineages (Schäfer et al. 2020), both developed by separate classical strain development programs.

Improvements such as high-copy insertions, elimination of unwanted side-activities, or deletions of proteases are introduced by genetic engineering (Punt et al. 2008; Heimel 2015), but the underlying chassis strains normally are older “working horses” of the relevant companies like Novonesis (formally Novozymes and Chr. Hansen), DSM, AB Enzymes, and others. Unfortunately, though understandably, publications from industry on these strains are rare, and studies on laboratory fungal strains are not easily transferable to industrial strains, particularly with regard to improvements in protein production. Furthermore, any effect on the cell wall composition or structure by high secretion or vice versa has until now not been a focus of research, and conclusions about its role are difficult to find in the literature.

In this section, we aim to highlight what has been investigated and published regarding the influence of cell wall mutants on enzyme production in industrially relevant filamentous fungi.

### Protein transfer through the fungal cell wall is influenced by biochemical interactions

Frequently, enzymes destined for secretion in their filamentous fungal production hosts appear to get stuck in the cell wall and are not released into the media as desired for proper downstream processing (Sato et al. 2011; Zhang et al. 2017a, b). This is more common for heterologous than for homologous proteins. As mentioned earlier, the permeability of filamentous fungal cell walls and the size of the secreted protein have a significant effect on whether it is released into the medium or retained in the periplasmic space (De Nobel and Barnett 1991). The cell wall can be considered a molecular sieve in which the hydrodynamic radius of a secreted molecule, rather than the molecular mass, is important. Hence, glycosylation, as a size increaser, influences transfer via the cell wall, but it also changes the polarity of a protein, and this can also affect the efficiency of proteins secretion into the medium (De Nobel and Barnett 1991). Such absorption effects of proteins and enzymes to the fungal cell wall have been reported for homologous alpha-amylase, heterologous bovine serum albumin, and hen egg lysozyme in *Aspergillus oryzae* (Sato et al. 2011). Recently, it was shown that a catalase produced in an industrially relevant *A. niger* strain was associated with the mycelium, whereas the production of glucoamylase consistently resulted in its secretion into the culture medium (Ram, unpublished data). Moreover, we recently found that two low-molecular-weight (only 20 kDa) luciferases, to be used as reporter genes for secretion, could not be found in the supernatant of *A. niger* cultivation. They remained associated with the biomass in their active, luminescence-producing forms (Ost and Dirks-Hofmeister, unpublished data). Cell walls thus can better be understood as gel matrices in which the density and degree of crosslinking but also the biochemical composition in terms of polarity and charge (e.g., content of chitosan or mannosylphosphates) define the porosity and permeability of secreted compounds (De Nobel et al. 1989, 1990b, 1990a). Clearly, cell-wall-trapped proteins might stick due to affinity reasons or get stuck because of steric hindrance, both limiting passage through the cell wall.

While it is difficult to change the biochemical characteristics of a target enzyme that is to be produced and secreted in filamentous fungi, a few attempts have shown that protein trapping can be circumvented by engineering cell wall-related genes in the production strain. Zhang et al. (2017a, b) reported that alpha-amylase (TAKA-amylase A, TAA) in *A. oryzae* binds to chitin or chitosan in the cell wall and that alpha-1,3-glucans can inhibit this absorption effect. Absorption – as binding of secreted enzymes to cell wall components – may result in the disappearance of enzyme products, especially during later stages of fermentation, and this is likely due to ionic interactions brought about by changes in the cell wall composition during the fermentation process (Sato et al. 2011). Early studies have shown that this binding is influenced by pH, chemicals, protease treatment, and other factors and that the binding of proteins can also be mediated by isolated cell walls (Tonomura et al. 1963; Yabuki and Fukui 1970).

### Productivity of filamentous fungi is related to macromorphology influenced by cell wall composition

Filamentous fungi exhibit diverse morphological phenotypes in submerged cultures that are particularly important for fermentability (reviewed by Papagianni 2004; Krull et al. 2013; Miyazawa et al. 2020; El Enshasy 2022). Macromorphology is known to be one major factor in the handling of filamentous fungi in industrial settings with large-scale bioreactor volumes but, more importantly, morphology seems to impact productivity (Grimm et al. 2005; Veiter et al. 2018). Fungal industrial enzyme-producing strains preferentially exhibit high-branching and dispersed growth phenotypes that enable lower viscosity, better mass transfer, and increased protein secretion (Wongwicharn et al. 1999; McIntyre et al. 2001; Wucherpfennig et al. 2010, 2011; Quintanilla et al. 2015).

The length of a hyphal element between two branching points is referred to as the hyphal growth unit (HGU) (Caldwell and Trinci 1973). A low HGU is preferred for industrial enzyme producers because the hyphal tip is assumed to be more permeable due to higher plasticity of the cell wall (Krull et al. 2013). Fungi growing in dense pellets show an increased number of inactive cores compared to viable hyphal tips, which limits mass transfer (Driouch et al. 2012). Additionally, Papagianni (2004) reviewed autoradiography studies from the 1970s, which showed progressive thickening of the cell walls located more distant from the apex. However, for some bulk chemicals, such as citric acid production in *A. niger*, pellet or clump morphology is advantageous (Papagianni 2004, 2007) while for others, such as penicillin production in *P. chrysogenum*, morphology does not seem to have a clear effect (Nielsen et al. 1995). Zheng et al. (2022) showed that overexpression of the catalytic subunit of cAMP-dependent protein kinase (*pkaC*) in *A. niger* altered pellet surface morphology and promoted hyphal growth at the surface. This leads to a looser outer layer, which improves the mass transfer of nutrients, oxygen, and secreted products, resulting in a 1.87-fold increase in citric acid production.

These examples, however, refer to small metabolites, in contrast to larger proteins and enzymes to be transferred via the cell wall, which could be the reason why morphology and a potentially corresponding cell wall have a stronger impact. A significant difference in the cell wall composition of a dispersed growing fungal strain in contrast to that of a pelleted growing strain seems plausible but has not yet been studied systematically. Kisser et al. (1980) reported a change in cell wall composition and morphology for citric acid production: the absence of manganese ions in the medium inhibited glycoprotein turnover and resulted in loss of hyphal polarity, increased branching, and chitin biosynthesis in the cell wall. In contrast, hyperbranching induced by deletion of *racA* (Rho-GTPase) in *A. niger* resulted in a modified number of hyphal tips (Kwon et al. 2011, 2014) and led to a four-fold increase in glucoamylase secretion (Meyer et al. 2011; Fiedler et al. 2018a). However, no analyses of the cell wall composition have been performed in these studies. Cairns et al. (2023) found that smaller pellets in *A. niger* were associated with higher protein titres. The analysis showed that a reduced pellet diameter and increased sensitivity to cell wall stress were positively correlated with increased protein secretion. Additionally, factors such as the radial growth rate and fitness under temperature or cell wall stress were described as key predictors of protein productivity. These findings suggest that manipulating pellet size and cell wall integrity could enhance protein yield in submerged cultures.

Further studies have reported targeted genetic modification of the cell wall to change the fungal macromorphology, thereby improving enzyme secretion. DSM patented an *agsE-*deleted strain of *A. niger* as an enzyme producer (Van Peij et al. 2014), and Novozymes (renamed Novonesis in 2023) followed shortly afterwards, stating that deleting more than one *ags*-gene was more beneficial than deleting *agsE* alone (Udagawa 2016). More recent studies on this particular alpha-glucan synthase have shown that its deletion is accompanied by dispersed growth of *A. niger* mutants in submerged cultures (Lyu et al. 2023; Ost et al. 2025). Furthermore, *agsB* is required for normal growth of *A. nidulans* under liquid culture conditions (Yoshimi et al. 2013) and the deletion of two chitin synthases significantly changed the morphology of *A. niger* (Müller et al. 2003). Thus, it can be concluded that the activity of cell wall-modifying enzymes and the macromorphology of filamentous fungi are causally related.

The question remains, if besides changes in morphology, changes in cell wall composition and/or increased enzyme secretion occur in morphological mutants. Some studies have used chitin synthase genes to alter the mycelial morphology to smaller, denser pellets and increase the product yields of penicillin in *P. chrysogenum* (Liu et al. 2013) or citric acid in *A. niger*, accompanied by a 50% reduction in chitin content in the cell wall (Sun et al. 2018). Previous studies have shown that chitin synthesis plays an important role in determining the hyphal morphology during submerged fermentation (Tsuizaki et al. 2013). Recently, Barthel et al. (2024) performed a systematic functional analysis of the entire predicted *chs* gene family (nine genes) in *A. niger*, which showed that the amounts of cell wall chitin and glucans, macromorphology, and protein secretion were affected by the deletion of specific *chs*-genes. Although the results showed that the deletion of *chsF* resulted in increased titers of extracellular proteins, the authors discussed that there was no obvious link between higher protein secretion and any specific phenotype that was determined in the study.

Deletion of all known *ags*-genes in *A. niger*, together with the whole *crh*-gene family which is involved in cross-linking chitin and glucans in the inner cell wall layer, also affects *A. niger* morphology in submerged cultures, although no changes in the monosaccharide composition of the cell wall or protein secretion have been observed (Ost et al. 2025). In contrast, Lyu et al. (2023) reported increased cellulase production in an *agsE* deletion strain. Moreover, an approach to increase the production of enzymes (cutinase encoding *cutL1*) in *A. oryzae* succeeded by deleting all three alpha-1,3-glucan synthases simultaneously, which decreased pellet size and was accompanied by increased productivity (Miyazawa et al. 2016). A follow-up study showed that combining these *ags*-mutations with the deletion of galactosaminogalactan synthesis resulted in a fully dispersed phenotype with improved fermentative culture rheology and increased productivity of recombinant cutinase (Miyazawa et al. 2019; Ichikawa et al. 2022).

Other reports have shown that enzyme production increases if the morphology is altered only by chemical means, for example, by the addition of inorganic microparticles (Driouch et al. 2011, 2012). Comparably, high agitation in a bioreactor leads to morphologically compact and strong mycelia and provides the cell wall with greater resistance to hydrolases in *A. niger* (Musílková et al. 1981).

These studies suggest that an altered cell wall composition can lead to changes in morphology. Although many morphological mutants have been described in the literature, only a few have analyzed cell wall or protein secretion. Thus, whether morphological changes always go along with changes in the cell wall composition remains to be investigated.

### High-secretion stress challenges cell wall integrity and potentially cell wall composition

As described earlier, enzyme secretion from the ER and Golgi apparatus via vesicle exocytosis is correlated with hyphal growth (Wösten et al. 1991; Kwon et al. 2011, 2014, 2017; Read 2011; Fiedler et al. 2018b, 2018a; Cairns et al. 2019b, 2021b, 2021a; Chevalier et al. 2023; Zuriegat et al. 2024). Jadhav et al. (2024) reviewed recently how high-secreting fungal strains are stressed due to their rapidly growing hyphal tips. This consequently not only feedback-stresses the ER machinery but also demands a constantly high supply of nutrients and oxygen with a suitable mass transfer to the inner cell machinery.

At the same time, endocytosis plays a key role in balancing secretion to sustain hyphal growth and cellular maintenance by retrieving excess membrane and recycling secretory components such as cell wall modifying enzymes (Higuchi et al. 2009; Steinberg et al. 2017; Commer and Shaw 2021; Barata-Antunes et al. 2021). Therefore, under high secretion stress, an increase in endocytosis may limit protein secretion efficiency by internalizing proteins before they reach their extracellular targets (Cairns et al. 2019b). During fermentation, secretion is no longer restricted to hyphal tips, but still requires dynamic rebalancing of exo- and endocytosis (Cairns et al. 2019b; Commer and Shaw 2021). Fungal morphology, such as hyphal branching and septation, must adapt to these changes to achieve higher productivity levels. A key regulator of protein secretion and hyphal growth has been identified in studies on the small GTPase Arf in *A. nidulans* and *A. niger* (Lee and Shaw 2008; Meyer et al. 2011; Fiedler et al. 2018b). ArfA is known to be involved in exocytic and endocytic pathways, and its overexpression leads to an increase in both total protein and glucoamylase secretion (Yahara et al. 2001; Meyer et al. 2011; Suda et al. 2018).

To circumvent ER stress, fungi like *Aspergillus* sp. have been shown to induce the expression of genes encoding ER-resident chaperones such as heat-shock proteins (Hsp 70, Hsp90, and Hsp60), foldases such as binding immunoglobulin protein (BiP), protein disulfide isomerase (PDI), calnexin (Clxa), calreticulin (Crt) or glucose-regulated proteins (Grp78), and numerous other proteins, including some involved in cell wall biosynthesis (Krishnan and Askew 2014; Lamoth et al. 2015; Zhang et al. 2017a, b; Rocha et al. 2020b). These proteins are involved in protein secretion as part of the unfolded protein response (UPR), which is initiated when the burden becomes too high to manage cellular homeostasis (reviewed by Heimel 2015; Cairns et al. 2019b). The secretory pathway appears to undergo almost complete restructuring in industrial strains to enhance the cellular architecture for efficient high-volume protein secretion. The interaction between the UPR and CWI pathways was reviewed by Malavazi et al. (2014) and is well discussed in further literature (Harting and Heimel 2020; Rocha et al. 2020a, 2020b). It has been mentioned that many cell wall components (proteins and most polysaccharides) depend on the ER machinery, such that cell wall damage also leads to ER stress and can activate the UPR response in fungi. The loss of UPR function causes cell wall defects in *S. cerevisiae* (Scrimale et al. 2009), and the same is likely to occur in filamentous fungi (Malavazi et al. 2014). This strong and bidirectional dependency of both pathways, UPR and CWI, clearly points to the cell wall, its integrity and composition being influenced by high-secretion stress. Furthermore, a changed cell wall could be a major prerequisite of fungi to enable UPR. Thus, studies on how the composition and/or crosslinking of the cell wall differs between UPR-stressed and non-stressed strains would be an interesting and relevant topic for future study.

In summary, providing a high secretion capacity for proteins in filamentous fungi demands a complex interaction from the ER machinery with adapted exo- and endocytosis, the CWI pathway maintaining cell walls, and the UPR ensuring proper folding. Thus, impaired responses may lead to cell clustering and reduced viability, which affect fermentation productivity. Consequently, these data suggest that understanding CWI and UPR coordination is essential for improving fungal protein secretion.

## Valorization of fungal biomass from biotechnological fermentation

We have already mentioned the enormous amounts of mycelia that accrue as by-products of fungal fermentations. Cell walls account for 20–40% of the dry weight of fungal mycelia (Lamon et al. 2023). Even if our estimated fungal fermentation volumes (Table 1) are partially derived from data with some uncertainties, our calculations still indicate that the estimated annual production of 67 million cubic meters would result in a conservatively estimated yield of six million tons of dry mycelium or two million tons of cell walls. If an average of 10% of this is chitin, which can be converted into more precious chitosans with a yield of approximately 75%, this would amount to 150,000 tons of chitosan annually. With a price tag of approximately 50 €/kg for reasonable quality chitosan, this would be a staggering 7.5 billion € – for the chitin alone! Most spent fungal biomass is used as low-value animal feed or even dumped onto fields as fertilizer, costing the producers a fee. Clearly, there are better uses for precious fungal biopolymers such as chitin and beta-glucans. Corresponding life cycle assessment studies would be very helpful and recommended as soon as the first solid data on resources and costs are available.

### Fungal cell walls as feedstock for the biotechnological production of biomolecules

A straightforward option for valorizing fungal cell walls from biotechnological fermentation wastes is to use them as feedstock for producing other commodities in bacteria or fungi. Yeast biomass, for example, a byproduct of beer brewing with *S. cerevisiae*, can be processed into yeast extracts that serve as food or feed additives and N-sources for microbial fermentation (Reed and Nagodawithana 1990; Jacob et al. 2019; Tao et al. 2023). In 1990, Reed and Nagodawithana reported that yeast cell wall mutants with altered morphologies were used by yeast extract manufacturers because they are highly sensitive to autolytic enzymes, but are also unstable, slow growing, and lyse even during growth. A comparable approach using filamentous fungal biomass as feedstock for microbiology is conceivable but has not been visibly discussed in the scientific literature. Yet, Abasian et al. (2020) showed that *Mucor rouxii* can grow and produce ethanol when the yeast extract in the medium is replaced by a fungal extract obtained by autolysis.

A relevant difference between yeast and filamentous fungi in this respect is the protein content of their biomass. Yeast extracts contain more than 60% protein, which provides accessible nitrogen for microorganisms (reviewed by Tao et al. 2023). The insoluble yeast cell wall fraction is often commercially sold as a dietary supplement under the term *glycan* (or beta-glucans) (Reed and Nagodawithana 1990). In contrast, filamentous fungal biomass after fermentation consists of a higher cell wall portion and a protein content lower than 20% (Isaza-Pérez et al. 2020), though it is noteworthy that specific filamentous fungi used for the production of single cell proteins (SCPs), also called Mycoproteins, may consist of 30–50% protein (Quorn™ as example with 45–54% protein content) (Ritala et al. 2017; Isaza-Pérez et al. 2020). Apart from its use as a nitrogen source, fungal extracts thus could be of interest as inexpensive C-sources (feedstock) for fermentation. It has already been shown that non-competitive bacterial consortia can be constructed to grow on chitin in pretreated fungal biomass and produce lysine (Vortmann et al. 2021). Depolymerization of glucans would even yield glucose as a more universal carbon source. In this respect beta-glucanase containing enzyme cocktails for yeast treatments like Glucanex®, VinoTaste® Pro, Snailase, Lyticase, Protplast F, and Yatalase are well known in the academic community of fungal genetics and biotechnology, as they are efficient for protoplast formation of filamentous fungi before transformation (De Bekker et al. 2009; Daly et al. 2017). A procedure that might enable the production of “fungal extracts” to be used in a manner similar to yeast extracts in biotech and microbiology.

In summary, we suggest that a process in which precious cell wall components (such as chitin) are first extracted and the remaining components of the fungal biomass are further processed (e.g., as feedstock for microorganisms) would be worth further elaboration. In particular, this would valorize and recycle a side-stream in the sense of a circular bioeconomy. To what extent the use of genetically modified strains as sources of the biomass/cell walls affects the potential utilization will likely not only depend on national regulations, but also on the treatment used (e.g. inactivation procedure, purification steps and/or DNA removal).

### Chitin/chitosan and beta-glucans are precious biopolymers in fungal cell walls that have applications, e.g., in medicine, food, feed, and agri- or horticulture

As described above, fungal cell walls contain diverse carbohydrate polymers. The most prominent one is chitin, whereas the most abundant ones are beta-glucans. There are many other homo- and heteropolymers containing, e.g., mannose, galactose, or galactosamine residues as monomeric building blocks, but these appear to be less significant in terms of their relative mass contribution to the cell wall, and their presence or absence strongly depends on fungal species and growth conditions.

Among glucans, which account for ca. 50–60% of cell wall dry weight, beta-1,3-glucans account for 65–90% of the total glucans (Bowman and Free 2006). It seems to be the one structural element that is indispensable for fungal vitality, and this is probably the reason why inhibitors of beta-1,3-glucan synthesis are the most successful antifungal drugs targeting fungal cell wall biosynthesis. (Ibe and Munro 2021). At the same time, beta-1,3-glucans are among the most prominent fungal pathogen-associated molecular patterns (PAMPs), which are recognized by cell surface receptors of the immune system of animals and humans (Mata-Martínez et al. 2022) and in the non-self-surveillance system of plants (Brown and Gordon 2001; Fesel and Zuccaro 2016). In animals, humans, and plants, both small linear beta-1,3-glucans (Klarzynski et al. 2000; Palma et al. 2006) and branched beta-1,3-beta-1,6-glucan heptamers (Sharp et al. 1984; Adams et al. 2008) appear to trigger immune responses, although the structural details of the PAMP-active oligomers differ. Clearly, such PAMPs have applications as immunostimulatory compounds, triggering “trained immunity” in animals and humans (Quintin et al. 2012) and defense priming in plants (Flors et al. 2024). In particular, branched beta-1,3-beta-1,6-glucan oligomers may offer promising options to support plant/animal/human vaccination strategies. In fact, “Zymosan,” a crude cell wall preparation from *S. cerevisiae* rich in beta-glucans, can be considered the role model for adjuvants in different types of vaccines (Fizpatrick and DiCarlo 1964; Nihei et al. 2023; Chin et al. 2025), and similar preparations, such as “Cerevisan,” are available to induce disease resistance in crop plants. However, such preparations contain, among other ingredients, a mixture of beta-glucans of different structures that induce different aspects of the immune system (Jin et al. 2018; Rainer et al. 2024). Structurally, slightly different beta-1,3-beta-1,6-glucans from filamentous fungi have been shown to act as either agonists or antagonists of their receptor, Dectin 1, in mouse and human cells (Smith et al. 2018). Similarly, of the hundreds of beta-1,3-beta-1,6-glucan oligomers, very few are elicitor-active in plants (Shibuya and Minami 2001; Ménard et al. 2004), and their activity varies dramatically with their molecular fine structure. We believe that this level of structural and functional detail for fungal beta-glucans, which far exceeds that from other sources, such as algae or bacteria, offers promising options for further optimizing the value chain from waste mycelial cell walls to functional biopolymers, as discussed below.

Besides glucans, chitin is also a PAMP that triggers immune reactions in humans, animals, and plants (Kaku et al. 2006; Bueter et al. 2013; Hembach et al. 2020). Structurally, chitin is simpler than glucan because it is a linear polymer of beta-1,4-linked *N*-acetylglucosamine residues, some of which may be deacetylated. Currently, most commercially produced chitin is converted into monomeric glucosamine (as the acetyl group is typically lost during chemical depolymerization) for use as an anti-arthritis food and feed supplement (D’Altilio et al. 2007; Conrozier and Lohse 2022), but some are partially deacetylated in concentrated hot alkali to yield chitosans (Younes and Rinaudo 2015). Chitosans are among the most versatile and promising functional biopolymers, which, depending on purity, can fetch prices of up to thousands of euros per kilogram (European Commission 2019; Huq et al. 2022). Clearly, this is an interesting and arguably the most interesting option for the valorization of mycelia waste from fungal fermentation.

However, can chitosan production from fungal chitin complement or compete with conventional chitosan production from crustacean waste? In terms of scale, we estimated that fungal fermentation waste could yield approximately 150,000 tons of chitosan annually. According to the United Nations Food and Agriculture Organization (FAO 2024), approximately 20 million tons of crustaceans were caught in 2022, which could theoretically yield perhaps 2 million tons of chitosan; however taking into account yields for the extraction and conversion steps (Muñoz et al. 2018), 500,000 tons is a more realistic number. While this is more than the estimated potential of fermentation mycelia, the actual global chitosan production from crustacean waste is probably less than ten percent of this potential, as logistics are difficult (Muñoz et al. 2018; Crognale et al. 2022). In addition, sustainability issues and the ecological footprint of chitosan production from crustacean waste have raised increasing concerns (Sebastian et al. 2020; Santos et al. 2020). This is where the sterile mycelial waste from large-scale fermentation with much less batch-to-batch variability may score points. Fungal chitosan is vegan, as compared to the animal origin of conventional chitosans, adding another plus in its favor for sensitive market sectors, such as cosmetics and food additives.

Another point worth mentioning is that true fungal chitosans – as opposed to chitosans produced by chemical deacetylation of fungal chitin – stand out. Some fungi contain natural chitosan in their cell walls, most prominently mucoromycete fungi, to which the industrially exploited *Rhizomucor* sp. and *Rhizopus* sp. belong (Table 1). These fungi enzymatically produce chitosan from chitin using chitin deacetylases (Jeraj et al. 2006; Gauthier et al. 2008; Lindner et al. 2025). To the best of our knowledge, it would seem that this natural chitosan differs in its acetylation pattern from chemically produced chitosans, which may make it unique in both structure and function (Cord-Landwehr and Moerschbacher, unpublished). In addition, the biotechnological production of natural chitosans may be more sustainable than conventional chemical chitosan production from chitin, but this can only be established through a thorough and comprehensive life cycle assessment (Muñoz et al. 2018).

There are many possible applications for chitosans, as mentioned in a plethora of reviews (Shariatinia 2019; Tang et al. 2023; Riseh et al. 2023, just to cite a few recent ones). Therefore, we will not go into detail here. Briefly, some chitosan-based products rely on the superior material properties of the biopolymers, such as their dye- and protein-binding and heavy metal-complexing abilities exploited for waste and drinking water purification or the finishing of papers and textiles (Pal et al. 2021). Here, purity demands – at least in terms of the absence of other biopolymers – may be less stringent than for products based on the biological functionalities of chitosans, such as their antimicrobial, plant-strengthening, or wound-healing activities (Jones et al. 2020; Eickelpasch et al. 2025). However, the latter products are potentially more attractive because chitosans can fetch better prices in these markets. The main problem with such products is that the bioactivities of chitosans critically depend on their molecular fine structure, most importantly their molecular weight and fraction of acetylation, and, as recently revealed, their acetylation pattern; however, structure–function relationships are still only partially understood. Recent advances in structural analysis and industrial production processes of chitosans have led to structurally and functionally well-characterized second-generation or 2G-chitosans with defined molecular weight and fraction of acetylation (Younes and Rinaudo 2015; Cord‐Landwehr et al. 2019; Wattjes et al. 2020; Hellmann et al. 2024). These are currently reviving the development of reliable chitosan-based products, such as biostimulants or biopesticides for agriculture or as wound dressings and drug, gene, or vaccine delivery systems in biomedicine. Current research is aimed at developing biotechnological processes for even better-characterized 3G-chitosans with not only tightly controlled molecular weight and fraction of acetylation, but also with defined, non-random acetylation patterns (Basa et al. 2020; Cord-Landwehr et al. 2020; Wattjes et al. 2020; Sreekumar et al. 2022; Singh et al. 2023; Hellmann et al. 2024; Richter et al. 2025). These are expected to offer even wider options for the development of chitosan-based products and applications. Clearly, with an optimistic outlook on future developments, the structural complexity of chitosans can become an asset rather than a problem.

This is where we see a similarity between chitosans and beta-glucans: Their structural complexity is the basis of their functional diversity. Once we understand better the structure–activity relationships of these precious biopolymers from fungal cell walls, we will be able to fine-tune their biological functionalities, producing the “next-generation” designer chitosans and beta-glucans specifically geared towards different uses and different markets. It is a challenge – but one well worth all efforts.

### The complexity of cell walls challenges and limits the valorization of filamentous fungal spent mycelia

Industrial-scale fungal chitin and glucan production is of economic interest because of its advantages such as year-round availability, controlled cultivation, and – at least presumed – reduced allergenic risks compared to crustacean-derived chitin. However, the main drawback of fungal chitosan is that chitin is covalently linked to glucans in the fungal cell wall, and potentially also to other components, such as proteins and melanin. Thus, extraction is difficult and purity is a problem. From scientific as well as patent literature, however, processes that show feasible extraction methods, such as alkali or acid treatment of fungal biomass, have been described (Dhillon et al. 2013; Islam et al. 2023). Patents from Cargill, Kitozyme SA, and others show that some industry sectors already have valorization of biomass from industrial fermented filamentous fungi on the horizon (Versali et al. 2002; Anderson et al. 2006; Banner et al. 2011; van Leeuwen et al. 2016).

While many publications report chitin extraction from fungal biomass (Fontaine et al. 2000; Heux et al. 2000; Wu et al. 2005), only some mention its purity. If at all, many studies have indicated the presence of glucose moieties in chitin or chitosan preparations, sometimes up to 50% of the dry weight. Clearly, this is not pure chitin or chitosan but rather a chitin/chitosan-glucan complex (Nwe and Stevens 2002). Depending on the use case, this does not have to be a problem (e.g., for applications relying on the polycationic nature of chitosan which is not impaired by the presence of glucans), but most of the time it will be (namely whenever the precise molecular characteristics of the chitin/chitosan need to be known to ensure reliable performance). However, fungal chitosans are increasingly appearing on the market, and some are surprisingly pure (Cord-Landwehr and Moerschbacher, unpublished).

Yet, to the best of our knowledge, attempts to increase the extractability of fungal biomass by cell wall mutations have not yet been described for filamentous fungi. It would be obvious to combine the advantages of fermentability, such as changes in morphology, improved fermentation performance, or reduced viscosity, as described for some cell wall mutants in the previous sections, with likely changes in the extractability of chitin/chitosan or beta-glucans. In addition to higher product yields and easier downstream processing, a more porous or loosely packed mycelial network could enhance the accessibility of chemical or enzymatic treatments used in the extraction of chitin and glucans from the biomass. One would assume that, in particular, modified crosslinks between chitin, glucans, and proteins in the fungal cell wall would likely enhance the efficiency of chitin and glucan extraction. Similarly, mutations that reduce the activity of chitin synthases or modify the acetylation pattern of chitin could also influence its solubility and extraction efficiency.

Overall, the use of cell wall mutants provides a promising strategy for improving the valorization of fungal biomass by making the extraction of key biopolymers more efficient and cost-effective. By integrating this approach into industrial fermentation processes for enzyme, protein, or metabolite production, fungal biomass will no longer be a mere byproduct, but a valuable resource for high-value biopolymer production. This circular bioeconomy model ensures that no fungal waste is discarded, thus reducing disposal costs and contributing to sustainable biomanufacturing in industrial biotechnology.

## Conclusion

Our literature review and analysis clearly highlight the relevance of cell walls in the biotechnological applications of filamentous fungi. Increasing knowledge of the composition, biosynthesis, and regulation of cell walls is continuously offering new possibilities to develop the cell wall machinery into a powerful set screw to optimize the biotechnological production of enzymes and metabolites. Cell walls play crucial roles in the transfer of proteins to the supernatant of fermentation media and determine fungal macromorphology, both of which have huge impacts on yield and productivity. A detailed understanding of these processes will allow for the design of targeted cell wall modifications by genetic modification and bioprocess engineering. However, the extent and limits of the influence of cell walls on the biotechnological productivity of filamentous fungal systems are yet to be elucidated.

Similarly, cell wall alterations by process and strain engineering offer huge potential for the improvement of valorization routes for fungal biomass as a side-stream of biotechnological production from precision fermentation. The ultimate challenge will be to combine both targets and improve efficiency in both production and valorization.

## Data Availability

The data supporting the findings of this study are available as cited in references. Unpublished data that are used as examples are available from the corresponding authors upon request.
